# Artificial ovaries constructed from biodegradable chitin-based hydrogels with the ability to restore ovarian endocrine function and alleviate osteoporosis in ovariectomized mice

**DOI:** 10.1186/s12958-023-01092-8

**Published:** 2023-05-19

**Authors:** Du Xiang, Encheng Zhou, Mei Wang, Kan Wang, Shujun Zhou, Qing Ma, Zibiao Zhong, Qifa Ye, Yun Chen, Xiaoli Fan, Yanfeng Wang

**Affiliations:** 1 Zhongnan Hospital of Wuhan University, Institute of Hepatobiliary Diseases of Wuhan University , Transplant Center of Wuhan University, Hubei Key Laboratory of Medical Technology on Transplantation, Wuhan, 430071 China; 2 Center for Reproductive Medicine, Zhongnan Hospital of Wuhan University, Wuhan, 430071 China; 3 Department of Radiation and Medical Oncology, Zhongnan Hospital of Wuhan University, Wuhan, 430071 China; 4 Department of Biomedical Engineering and Hubei Province Key Laboratory of Allergy and Immune Related Diseases, TaiKang Medical School (School of Basic Medical Sciences), Wuhan, 430071 China

**Keywords:** Artificial ovary, 3D culture, Follicle, Alginate, Ovariectomised, Chitin

## Abstract

**Background:**

Artificial ovary (AO) is an alternative approach to provide physiological hormone to post-menopausal women. The therapeutic effects of AO constructed using alginate (ALG) hydrogels are limited by their low angiogenic potential, rigidity, and non-degradability. To address these limitations, biodegradable chitin-based (CTP) hydrogels that promote cell proliferation and vascularization were synthesized, as supportive matrix.

**Methods:**

In vitro, follicles isolated from 10–12-days-old mice were cultured in 2D, ALG hydrogels, and CTP hydrogels. After 12 days of culture, follicle growth, steroid hormone levels, oocyte meiotic competence, and expression of folliculogenesis-related genes were monitored. Additionally, follicles isolated from 10–12-days-old mice were encapsulated in CTP and ALG hydrogels and transplanted into the peritoneal pockets of ovariectomised (OVX) mice. After transplantation, steroid hormone levels, body weight, rectal temperature, and visceral fat of the mice were monitored every two weeks. At 6 and 10 weeks after transplantation, the uterus, vagina, and femur were collected for histological examination.

**Results:**

The follicles developed normally in CTP hydrogels under in vitro culture conditions. Additionally, follicular diametre and survival rate, oestrogen production, and expression of folliculogenesis-related genes were significantly higher than those in ALG hydrogels. After one week of transplantation, the numbers of CD34-positive vessels and Ki-67-positive cells in CTP hydrogels were significantly higher than those in ALG hydrogels (P < 0.05), and the follicle recovery rate was significantly higher in CTP hydrogels (28%) than in ALG hydrogels (17.2%) (P < 0.05). After two weeks of transplantation, OVX mice implanted with CTP grafts exhibited normal steroid hormone levels, which were maintained until week eight. After 10 weeks of transplantation, CTP grafts effectively ameliorated bone loss and atrophy of the reproductive organs, as well as prevented the increase in body weight and rectal temperature in OVX mice, which were superior to those elicited by ALG grafts.

**Conclusions:**

Our study is the first to demonstrate that CTP hydrogels support follicles longer than ALG hydrogels in vitro and in vivo. The results highlight the clinical potential of AO constructed using CTP hydrogels in the treatment of menopausal symptoms.

**Supplementary Information:**

The online version contains supplementary material available at 10.1186/s12958-023-01092-8.

## Background

After menopause, women experience various physiological and psychological symptoms, such as atrophy of the reproductive organs and osteoporosis [1, 2]. Although traditional pharmacologic hormone replacement therapy (pHRT) is beneficial in healthy younger women (aged 50–59 years), it increases the risk of stroke, venous thromboembolism, and breast cancer [3, 4]. In this context, artificial ovaries (AOs) offer a promising solution for maintaining physiological hormonal levels in women with ovarian dysfunction [5]. AOs consist of follicles, growth factors, and ovarian stromal cells encapsulated in a scaffold to simulate the functions of natural ovaries [6, 7]. The primary challenge in this approach is the construction of a biodegradable and biocompatible scaffold that encapsulates isolated follicles and supports their survival and growth after transplantation [8]. Alginate (ALG), a linear polysaccharide generated by brown algae and bacteria, is the most widely used scaffold in fabricating AOs [9]. ALG hydrogels cross-linked with calcium under physiological conditions provide a 3D structural matrix for the development of follicles; however, the rigidity, low angiogenic potential, and non-degradability of ALG hydrogels limit their use in AO applications [10, 11].

The secondary follicles with an initial diametre of 120 μm can grow into mature follicles with diametre of 400 μm, an approximate 37-fold increase in volume [12]. Therefore, biological scaffolds must accommodate this substantial volumetric expansion of follicles. As follicular growth is regulated by the mechanical properties of the ovarian environment, matrix stiffness is mechanically important for maintaining normal follicular growth [13, 14]. The Young’s modulus values considered permissive for follicle growth range from 100 to 800 Pa [14]. A transplantable AO should ensure favourable cell proliferation and differentiation, cell invasion and vessel recruitment; thus, creating an optimal environment for the growth and survival of follicles owing to gradual matrix degradation [15]. Insufficient neovascularisation after transplantation of AOs is a major obstacle that causes a large number of follicles to degenerate owing to ischemia and hypoxia [16]. Therefore, it is necessary to accelerate graft revascularisation and reduce hypoxia, particularly during the early post-grafting period.

Chitin is the second most abundant natural polymer. Chitin-based scaffolds exhibit properties such as degradability, biocompatibility, and mechanical strength, which are necessary in tissue engineering applications [17]. Chitin is widely used in regenerative medicine, as it facilitates the recruitment of endothelial cells, thereby contributing to angiogenesis [18, 19]. Therefore, owing to its beneficial properties, chitin is a potentially promising candidate for the construction of AOs. In the present study, to increase vascularisation and improve the therapeutic effects of AOs, adipic dihydrazide-grafted carboxyethyl chitin (CECT-ADH) /tert-butyl acetoacetate-grafted poly (vinyl alcohol) (PVA-AA) (CTP) hydrogels were synthesized using acylhydrazones, according to the 3D-hydrogel criteria [12, 20]. Compared with ALG, CTP hydrogels degrade in response to an increase in volume. Furthermore, the Young’s modulus of 0.5% ALG hydrogels is 300 Pa as previously reported [13], whereas that of CTP hydrogels is 228 Pa. Subsequently, we investigated whether the fabricated AOs could alleviate oestrogen deficiency-related symptoms in ovariectomised (OVX) mice. To the best of our knowledge, this is the first study to report the construction of AOs using chitin-based hydrogels. The findings presented in this study could provide a framework for the application of AOs in the clinical treatment of menopausal symptoms.

## Methods

### Study design

Female BALB/c mice (10–12 days old and 8–10 weeks old) were purchased from the Center for Animal Experiments at Wuhan University (Wuhan, China). The mice were housed in a temperature-controlled (25℃) and humidity-controlled (40%) animal room with a 12 h light/dark cycle and free access to food and water. All surgeries were performed intraperitoneally using 40 mg/kg sodium pentobarbital as the anesthetising agent. Mice had similar initial mean body weights and rectal temperatures. Body weights and rectal temperatures were monitored using a Digital balance (Jiangsu, Tong Jun) and thermal probe (1529 thermometer, Fluke Corporation, USA), respectively, every 2 weeks. The vaginal cytology of mice was examined daily at approximately 9:00–10:00 AM.

### Hydrogel preparation

Sodium alginate (55–65% guluronic acid) was purchased from Sigma-Aldrich (St. Louis, MO, USA). The ALG hydrogel was prepared as previously described by West et al. [21]. Chitin powder (molecular weight: 3 ⋅ 10^5^ g/mol, degree of acetylation: 92%) was purchased from Golden-Shell Biochemical (Zhejiang, China) and purified as previously described [22]. CECT-ADH was synthesized as previously described [23]. PVA-AA was synthesized as previously described, with slight modification [24]. Briefly, a three-necked flask was placed in an oil bath at 80 °C and charged with PVA in a dry nitrogen atmosphere. Thereafter, dimethyl sulphoxide was introduced into the flask via a syringe, and the mixture was stirred until completely dissolved. Subsequently, tert-butyl acetoacetate was introduced dropwise, and the reaction was performed for 4 h. The resulting mixture was precipitated in ethyl ether and dialyzed (MWCO 3.5 KDa) against 25% ethanol aqueous solution and distilled water. CECT-ADH/PVA-AA hydrogels (CTP hydrogels) were fabricated via acylhydrazone by mixing CECT- ADH solution and PVA-AA solution (1:1). The characterisation of CTP hydrogels has been presented in other study.

### Follicle isolation, encapsulation, and culture

The ovaries of 10–12-days-old mice were removed and transferred to α-minimal essential medium (MEM) containing 5% fetal bovine serum (FBS; Gibco, UK). Under a stereomicroscope (Olympus, SZX16, Japan), intact preantral follicles (90–100 μm) were mechanically isolated using 28 G syringe needles. A single follicle was transferred into 5 µL 0.5% ALG and immersed in a solution containing 50 mM CaCl_2_ and 140 mM NaCl for 30 s for the crosslinking reaction to occur. For the preparation of CTP hydrogels, droplets of 5 µL of PVA–AA were introduced on 96-well plates, and a single follicle in 5 µL of CECT–ADH was mixed with PVA–AA. CTP hydrogels were cross-linked for 5 min at 37 °C in an incubator. After encapsulation, the follicles were plated in 96-well plates containing 100 µL growth medium and cultured for 13 days as previously described [21]. Every two days, 50 µL of the medium was replaced, and the resulting conditioned medium was stored at − 80 °C for hormonal assays.

### Morphological and survival assessment of follicles

On days 0, 6, and 12 of the follicle culture periods, the morphology of follicles was imaged and observed using an inverted microscope, and ImageJ software was used to measure the follicle diametre (n = 54 for each group). Follicles were classified as morphologically normal or degenerated as previously described [25].

### Assessment of steroid hormones

Blood samples were centrifuged at 3000 rpm for 15 min at 4 °C to obtain serum samples. Androstenedione, 17β-oestradiol, and progesterone levels in the medium and serum were measured using commercially available ELISA kits (Cloud-Clone Corp, Wuhan, China). The kit sensitivities were 44.2 pg/mL (CEA456Ge), 4.45 pg/mL (CEA461Ge), and 0.47 ng/mL (CEA459Ge), respectively.

### Follicle viability assay

After follicle isolation, a total of 20 preantral follicles were transferred to 1 mL of phosphate buffered saline containing 2 µM of calcein-AM and 5 µmol/L of ethidium homodimer (Molecular Probes, St. Louis, MO, USA). Follicles were incubated with fluorescent dyes for 30 min at 37℃ in the dark.

### Oocyte meiotic competence

Oocyte meiotic competence was assessed as previously described, with slight modifications [13]. Briefly, antral follicles were removed from the hydrogels using alginate lyase and lysozyme. Thereafter, the follicles were cultured in a maturation medium (α-MEM supplemented with 10% FBS, 5 ng/mL epidermal growth factor, 1.5 IU/mL human chorionic gonadotropin, and 10 mIU/mL FSH) for 16–18 h. After incubation, the oocytes were separated from the surrounding cumulus cells by treatment with 0.3% hyaluronidase. The oocyte state was assessed using light microscopy and characterised as previously described [26].

### Real-time polymerase chain reaction (RT-PCR)

To evaluate the expression of folliculogenesis-related genes, follicles from all groups were collected on culture day 12 (30 follicles/replicate). Quantitative RT-PCR assays were performed as previously described [27]. The sequences of primers are presented in Table 1.

### Encapsulation of enzymatically isolated follicles and stromal cells for AO transplantation

For animal experiments, ovaries were excised from 10–12-days-old mice and enzymatically digested as previously described [15]. After isolation, 10 µL of CECT-ADH was pipetted into the concentrated follicle suspension (1 µL) and gently mixed with 10 µL of the PVA-AA crosslinker. Groups of 100 follicles were encapsulated in CTP hydrogels to construct AOs sized 5 mm × 5 mm for transplantation, as described in Fig. S1a. 100 follicles were pipetted into the middle of each ALG droplet (20 µL), and the droplets were slowly introduced into the chemical crosslinking solution and crosslinked for 2 min. The prepared grafts were immediately transplanted to the peritoneal pockets of the 10-weeks-old OVX mice.

### Ovariectomy and AO transplantation

A total of 48 8–10-weeks-old mice underwent bilateral ovariectomy after anesthesia. A total of 12 sham mice underwent only bilateral incisions. After at least 14 days, the mice were randomly assigned to five groups (n = 12): sham (ovaries intact), OVX (bilateral ovariectomies), CTP-F (bilateral ovariectomies, chitin hydrogel without follicles), ALG (bilateral ovariectomies, ALG hydrogels with follicles), and CTP (bilateral ovariectomies, chitin hydrogels with follicles) groups. Mice were sacrificed at 6 (n = 30) and 10 (n = 30) weeks after transplantation. To induce angiogenesis, a circular pocket was created on the inner side of the peritoneum (Fig. S1b). After the grafts were gently slid into the peritoneal pocket, the AOs were transplanted onto each side (Fig. S1c). After 10 weeks of grafting, blood vasculature was apparent around the CTP grafts, as indicated by the dotted circle in Fig. S1d.

### Organ index

After the mice were sacrificed, the uterus, vagina, and visceral fat were excised and weighed, and the organ index was calculated according to the ultimate body weight of the mice.

### Haematoxylin and eosin (H&E) staining

After mechanical isolation, 10 follicles were covered with 2% melted agarose and immediately fixed in 4% formalin for histological evaluations. Following sacrifice, the grafts associated with encompassing tissue, uterine tissue, vaginal tissue, and right femurs of the mice were excised and immediately fixed in 4% formalin. The samples were sectioned at 5-µm thickness and stained H&E. The number of follicles was counted as previously described [15]. Follicle counts were performed by an experienced researcher blinded to experimental conditions. The follicle recovery rate was determined by the ratio of the number of recovered follicles to the number of follicles grafted. Vaginal epithelial thickness and uterine endometrial thickness were calculated as previously described [28]. Additionally, the number of vaginal epithelial cellular layers was counted as described by Huang et al. [29].

### Micro-computed tomography (CT) scanning and analysis

The left femurs of the sacrificed mice were fixed in 4% buffered formalin. Micro-CT scanning and analysis were performed as previously described [30].

### Immunohistochemistry

Immunohistochemical staining for CD34, Ki-67, and CD45 was performed to characterise the isolated follicles for grafting. The protocol was performed as previously described by Herraiz et al. [31]. Antibodies against CD34 (1:100 dilution, ab81289, Abcam, UK), Ki-67 (1:200 dilution, ab279657, Abcam, UK), and CD45 (1:200 dilution, ab10558, Abcam, UK) were used. Integrated optical density was calculated using ImageJ software.

### Follicle apoptosis assay

Apoptosis was analysed using a terminal deoxynucleotidyl transferase-mediated dUTP nick end labelling (TUNEL) assay to detect DNA fragmentation with an In Situ Cell Death Detection Kit TMR Red (Roche, China). The protocol was performed as previously described [32].

### Degradation test

Homogenous hydrogels were prepared and subcutaneously implanted into the backs of mice. At the end of 10 weeks, blood samples and major organs were stained using H&E to assess biocompatibility.

### Statistical analysis

Statistical analysis was performed using the SPSS software (version 22.0; SPSS, Inc.). The results of different groups were compared using one-way ANOVA followed by Tukey’s post-hoc test and are expressed as the mean ± standard deviation. Categorical data were analysed using the χ2 test for independent samples. Differences were considered statistically significant at P < 0.05.

**Table 1:** The characteristic of primer sequences used in real-time quantitative reverse transcription polymerase chain reaction assays

Genes	Primer sequence (5’-3’)	GenBank accession numbers	Product size (bp)
FSH-R	F:ACAGGGTCTTCCTCTGCCAA R:GGTTGGAGAACACATCTGCC	14,309	201
CYP19a1	F:CGCAGAGTATCCAGAGGTCG R:CGCATGACCAAGTCCACAAC	13,075	159
AMH	F:TTTGGTGCTAACCGTGGACT R:CAGCGGGAATCAGAGCCAAA	11,705	138
PCNA	F:GCACGTATATGCCGAGACCTT R:GACAGTGGAGTGGCTTTTGT	18,538	233
BMP15	F:CATCCAAGGGAGAACCGCAC R:AGTTGATGGCGGTAAACCACA	12,155	176
GAPDH	F:ACTCTTCCACCTTCGATGCC R:TGGGATAGGGCCTCTCTTGC	14,433	193
CYP11a1	F:TATATTGGGCTGGGCAAGTGTTA R:AAAGTGCCCAGCTTCTCCCTATA	13,070	236
CYP17a1	F:GGCTAACATTGACTCCAGCATTG R:CTGGGTGTGGGTGTAATGAGATG	13,074	171
STAR	F:AGCATGTTCCTCGCTACGTT R:ACCTCTCCCTGCTGGATGTA	20,845	242
HSD3B1	F:AGATGTTGGTGCAGGAGAAAGAA R:CAACACTGTCACCTTGGTCTTTG	15,492	116

## Results

### Follicle structure and vitality

**Fig. 1:**
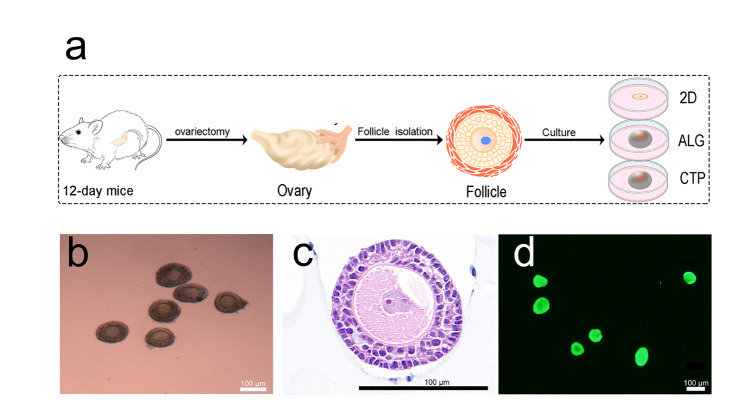
Structure and viability of the follicles. (a) Schematic illustration of in vitro culture. (b) Light microscopy images of isolated follicles. (c) Isolated follicle stained with hematoxylin and eosin. (d) Follicular viability assessment by staining with calcein-AM and ethidium homodimer-I

A schematic illustration of the in vitro culture (IVC) method used in this study is depicted in Fig. 1a. Light microscopy images of the isolated follicles are presented in Fig. 1b, demonstrating that healthy follicles are composed of oocytes and somatic cells. Individual isolated follicles stained with H&E are shown in Fig. 1c, confirming the structural integrity of the follicles. Figure 1d shows that the isolated follicles survived after mechanical isolation. Conclusively, our results revealed that mechanical isolation does not damage follicle structure and viability, and follicles isolated in this manner can be used for IVC and transplantation.

### Follicle growth and steroid production in vitro

**Fig. 2:**
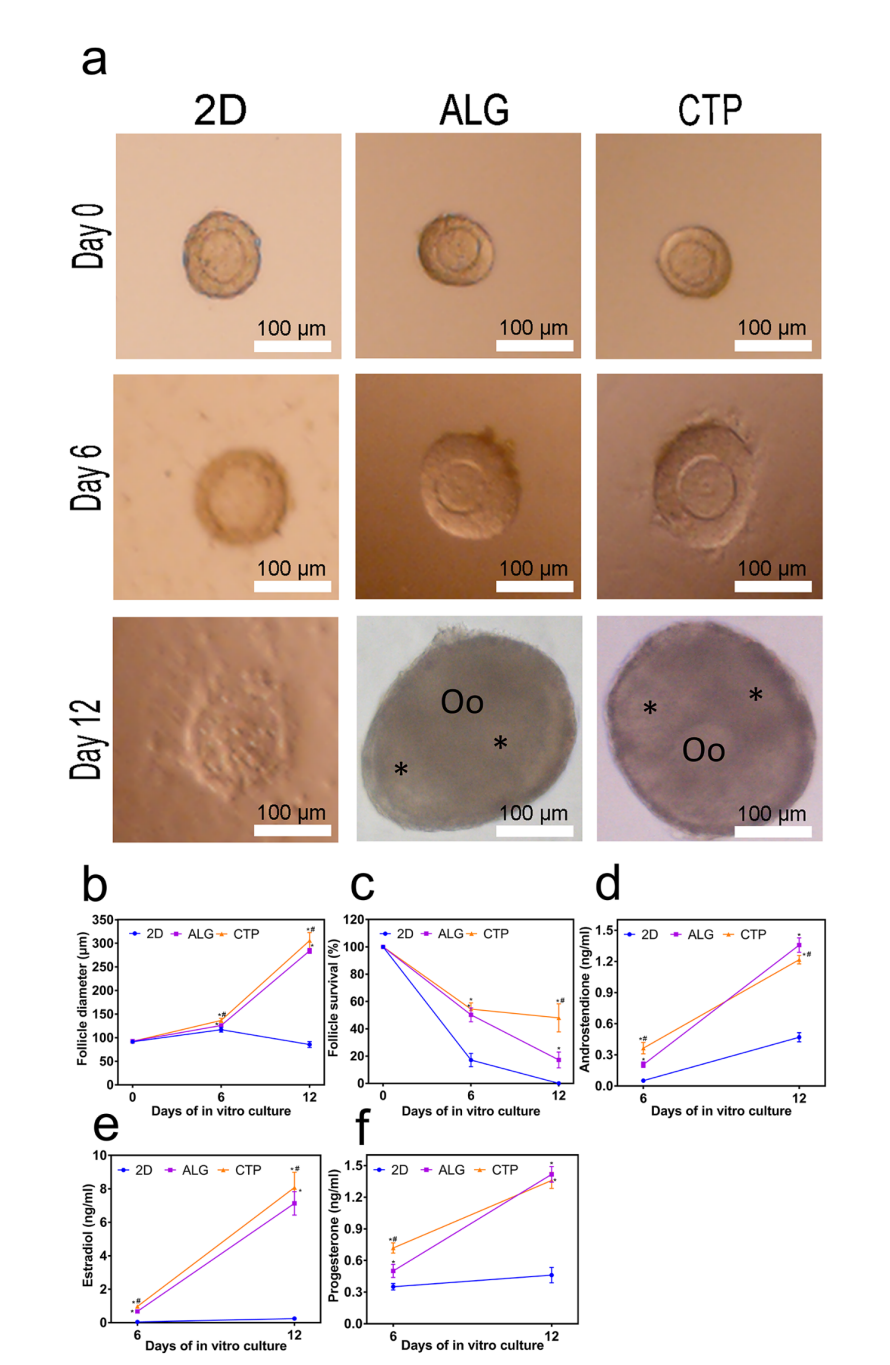
Growth and hormone secretion. (a) Morphology of follicles cultured from days 0–12. (b) Growth curve over a 12-day culture period. (c) Follicular survival rate in vitro measured on days 6 and 12. (d-f) Androstenedione, oestradiol, and progesterone levels secreted into the culture medium on days 6 and 12. The data are represented as the mean ± standard deviation. * indicates significance at P < 0.05 compared with 2D, and # indicates significance at P < 0.05 compared with alginate hydrogels (ALG) determined using one-way ANOVA, followed by Student–Newman–Keuls post-hoc analysis

Morphologically normal follicles were cultured after isolation. Light microscopy showed that the follicles had intact spherical structures in the ALG and CTP groups during IVC (Fig. 2a). A fluid-filled antrum cavity was observed on day 12 in both the ALG and CTP groups, consistent with the in vivo morphology. Conversely, the link between oocytes and granulosa cells (GCs) was broken in the 2D groups because the GCs adhered to the bottom of the culture dish, resulting in eventual follicular damage (Fig. 2a). The follicle diametres in the CTP groups on days 6 and 12 were significantly larger than those in the ALG groups (P < 0.05) (Fig. 2b). Moreover, the survival rate of follicles in the CTP groups was significantly higher than that in the ALG groups on day 12 of IVC (P < 0.05) (Fig. 2c), suggesting that CTP hydrogels could support follicles for longer duration than ALG hydrogels in vitro.

Ovarian hormones, including androstenedione (A), oestradiol (E), and progesterone (P), are secreted by theca cells and GCs. The levels of A in the ALG groups were significantly lower than that in the CTP groups on day 6 of IVC but higher than that in the CTP groups on day 12 of IVC (P < 0.05) (Fig. 2d). Levels of E in the CTP groups were significantly higher than those in the ALG groups on days 6 and 12 (P < 0.05) (Fig. 2e). Levels of P in the ALG groups were significantly lower than those in the CTP groups on day 6 of IVC (P < 0.05) but higher than those in the CTP groups on day 12 of IVC (P > 0.05) (Fig. 2f). These results imply that follicular growth was supported in the CTP hydrogels, and more oestrogen was produced in the CTP groups than in the ALG groups.

### Characterisation of differential gene expression using RT-PCR and oocyte meiotic competence

**Fig. 3:**
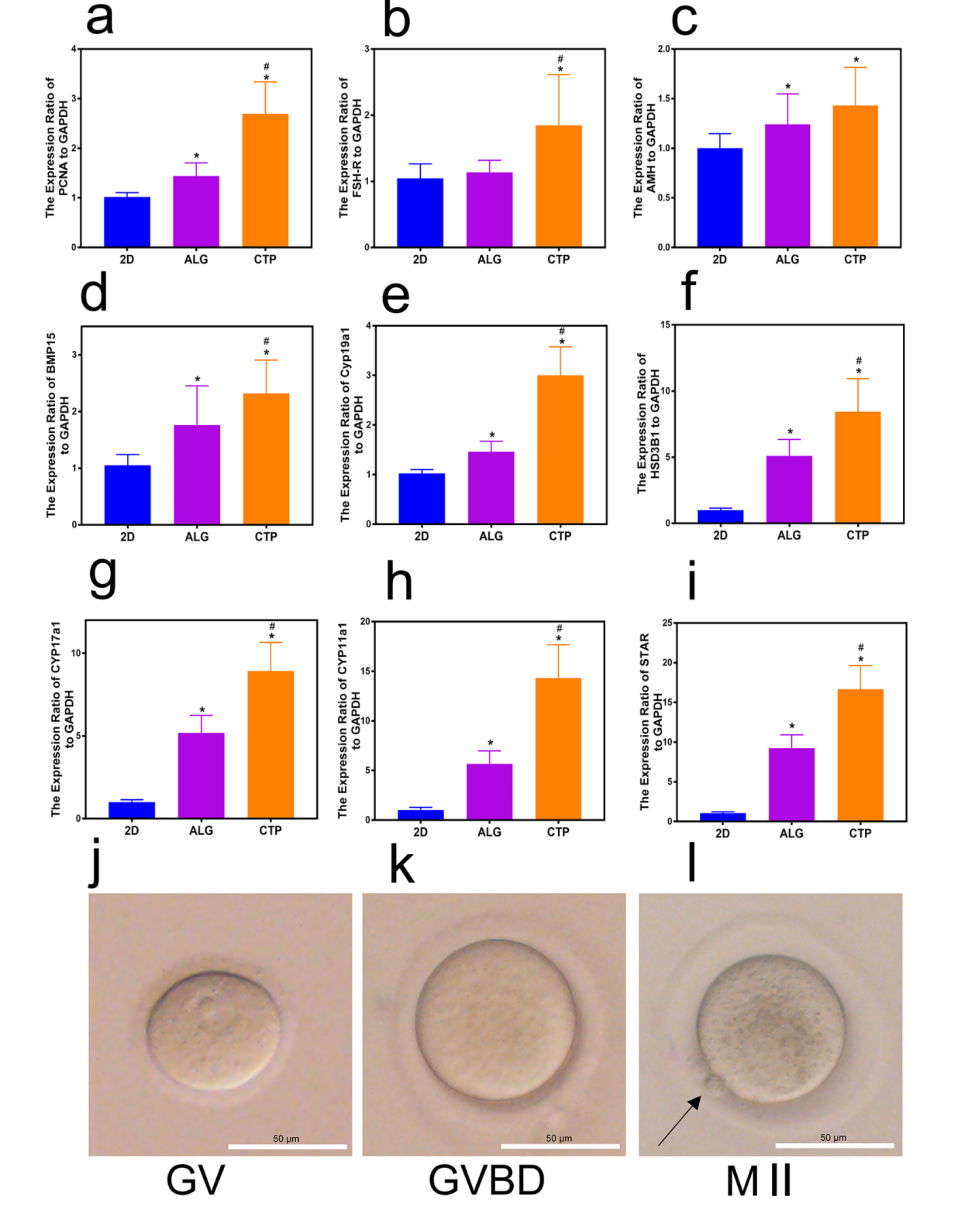
Real-time polymerase chain reaction and oocyte meiotic competence. (a-i) Analysis of *PCNA*, *FSH-R*, *AMH*, *BMP15*, *CYP19a1*, *HSD3B1*, *CYP17a1*, *CYP11a1*, and *STAR* expression levels on day 12 of culture. (j-l) Representative images of oocyte stages after in vitro follicle maturation in CTP hydrogels. GV germinal vesicle in a mature oocyte (j) GVBD germinal vesicle breakdown (k), The first polar body (arrow) was extruded in metaphase II (M II) (l). GAPDH was used as the internal control. The data are represented as the mean ± standard deviation. * indicates significance at P < 0.05 compared with 2D, and # indicates significance at P < 0.05 compared with alginate hydrogels (ALG) determined using one-way ANOVA, followed by Student–Newman–Keuls post-hoc analysis

At the end of the culture period, the expression levels of folliculogenesis-related genes were assessed. PCNA, FSH-R, and AMH are considered key markers of GCs. Figure 3a-c shows that the expression levels of PCNA, FSH-R, and AMH were higher in the CTP groups than those in the other two groups, indicating that the CTP groups contained more GCs (P < 0.05). BMP15 is an oocyte-secreted factor crucial for regulating follicular growth [27, 33]. Our findings showed that mRNA expression of BMP15 was higher in the CTP groups than in the ALG groups (Fig. 3d). Cyp19a1, HSD3B1, CYP17a1, and CYP11a1 are important enzymes that convert androgen to oestrogen; the higher the activity of these enzymes, the more oestrogen is produced [34]. Figure 3e-h demonstrate that the expression levels of Cyp19a1, HSD3B1, CYP17a1, and CYP11a1 were higher in the CTP groups than those in the other two groups, consistent with the results shown in Fig. 2e. STAR is known to be responsible for transporting cholesterol and providing substrates for oestrogen production [35]. Figure 3i shows that the expression of STAR mRNA in the CTP groups was higher than that in the other two groups (P < 0.05). These results reveal that the CTP hydrogels are more suitable for follicular growth, as indicated by increased GC proliferation, hormone secretion, and steroidal aromatase activity.

Subsequently, the quality of oocytes obtained from follicles cultured in hydrogels was measured based on their ability to resume meiosis. Follicles encapsulated in CTP hydrogels could resume meiosis and produce meiotically competent oocytes, as shown in Fig. 3j-k. Moreover, the percentage of M II stage oocytes obtained from follicles cultured in CTP hydrogels (78.9%) was significantly higher than that cultured in ALG hydrogels (56.7%), as shown in Table 2.

**Table 2:** Oocyte meiotic competence

Conditions	Oocytes, n	M II, n (%) ^a^	GVBD, n (%)	GV, n (%)	DG, n (%)
ALG	37	17 (56.7%) ^b^	30 (81.1%)	3 (8.1%)	4 (10.8%)
CTP	43	30 (78.9%) ^c^	38 (88.4%)	2 (4.7%)	3 (7%)

^a^ The percentage of M II eggs was calculated relative to GVBD oocytes

^b−c^ Different superscripts within each column indicate statistically significant differences (P<0.05)

DG, degenerated; GV, germinal vesicle; GVBD, germinal vesicle breakdown; M II, metaphase II.

### Evaluation of the grafts post-transplantation

**Fig. 4:**
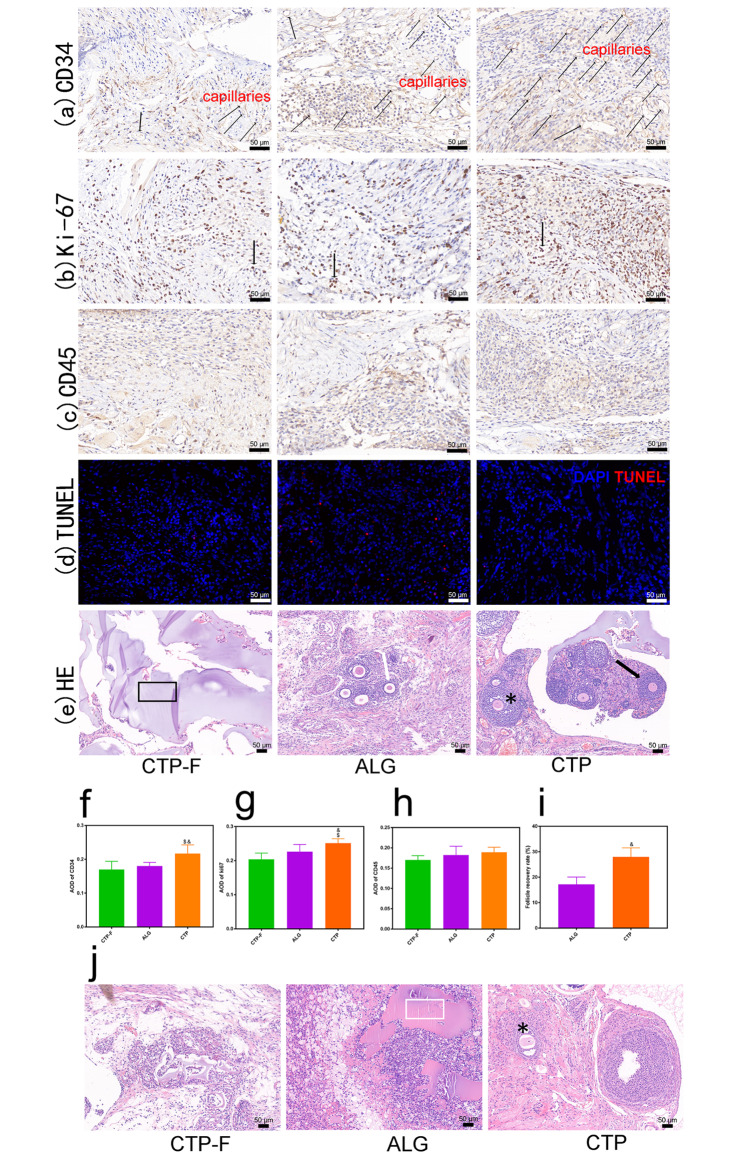
Evaluation of the grafts post-transplantation. (a) Blood vessels and endothelial cells stained with CD34; black arrows indicate capillaries. (b) Ki67-positive cells were stained brown (black arrows). (c) CD45-positive cells observed in the grafts. (d) Terminal deoxynucleotidyl deoxyuridine triphosphate nickend labeling (TUNEL) assay illustrating apoptotic cells. Red fluorescence: TUNEL-positive nuclei; blue fluorescence: DAPI. (e) Representative images of haematoxylin and eosin (H&E) staining of the grafts at 1 week after transplantation. Primary follicles (white arrows), secondary follicles (black arrows), antral follicles (asterisk), and CTP hydrogels (black rectangles) were observed. Average optical density values of CD34 (f), Ki-67 (g), CD45 (h), and follicle recovery rate (i) were quantified. (j) Representative images of H&E staining of the grafts at 10 weeks after transplantation. Antral follicles (asterisk) and ALG hydrogels (white rectangle) were observed. $ indicates significance at P < 0.05 compared with the CTP-F groups; & indicates significance at P < 0.05 compared with the ALG groups, determined using one-way ANOVA, followed by Student–Newman–Keuls post-hoc analysis

After 1 week of grafting, primary, secondary, and antral follicles were observed in ALG and CTP groups (Fig. 4e). Follicular recovery rate on day seven in CTP hydrogels was 28% (42 normal follicles out of 150 grafted follicles), compared to 17.2% (25 normal follicles out of 145 grafted follicles) in ALG hydrogels, showing a significant difference between the two groups (χ2 = 4.862, P = 0.027) (Fig. 4i). After 10 weeks of grafting, antral follicles were observed only within the CTP groups and not in the ALG groups (Fig. 4j). Moreover, large amounts of undegraded ALG hydrogels were observed, as indicated by the light pink stain; however, only a small amount of undegraded CTP hydrogels were observed (Fig. 4j).

In this study, CD34 and Ki-67 levels were used as indicators of revascularisation and cell proliferation, respectively. Figure 4a shows that the average optical density (AOD) of CD34 in the CTP groups was higher than that in the ALG groups after 1 week of transplantation (P < 0.05). Moreover, the Ki-67 staining results showed significantly increased cell proliferation in mice transplanted with CTP grafts compared to those with ALG grafts (Fig. 4b). CD45 immunostaining was used to evaluate the healing process after injury induced by scraping the peritoneal surface of the mice. No fibrous capsules were observed around the grafts, and CD45-positive cells were identified in the grafts (Fig. 4c); the three groups showed no significant differences. Results of the TUNEL assay showed that a small number of apoptotic cells appeared in all three groups, with fewer apoptotic cells in the CTP groups than those in the ALG groups (Fig. 4d). Conclusively, our results demonstrated that the CTP matrix is biodegradable and can promote angiogenesis and cell proliferation.

### Hormone levels and their effects on body composition after AO transplantation

**Fig. 5:**
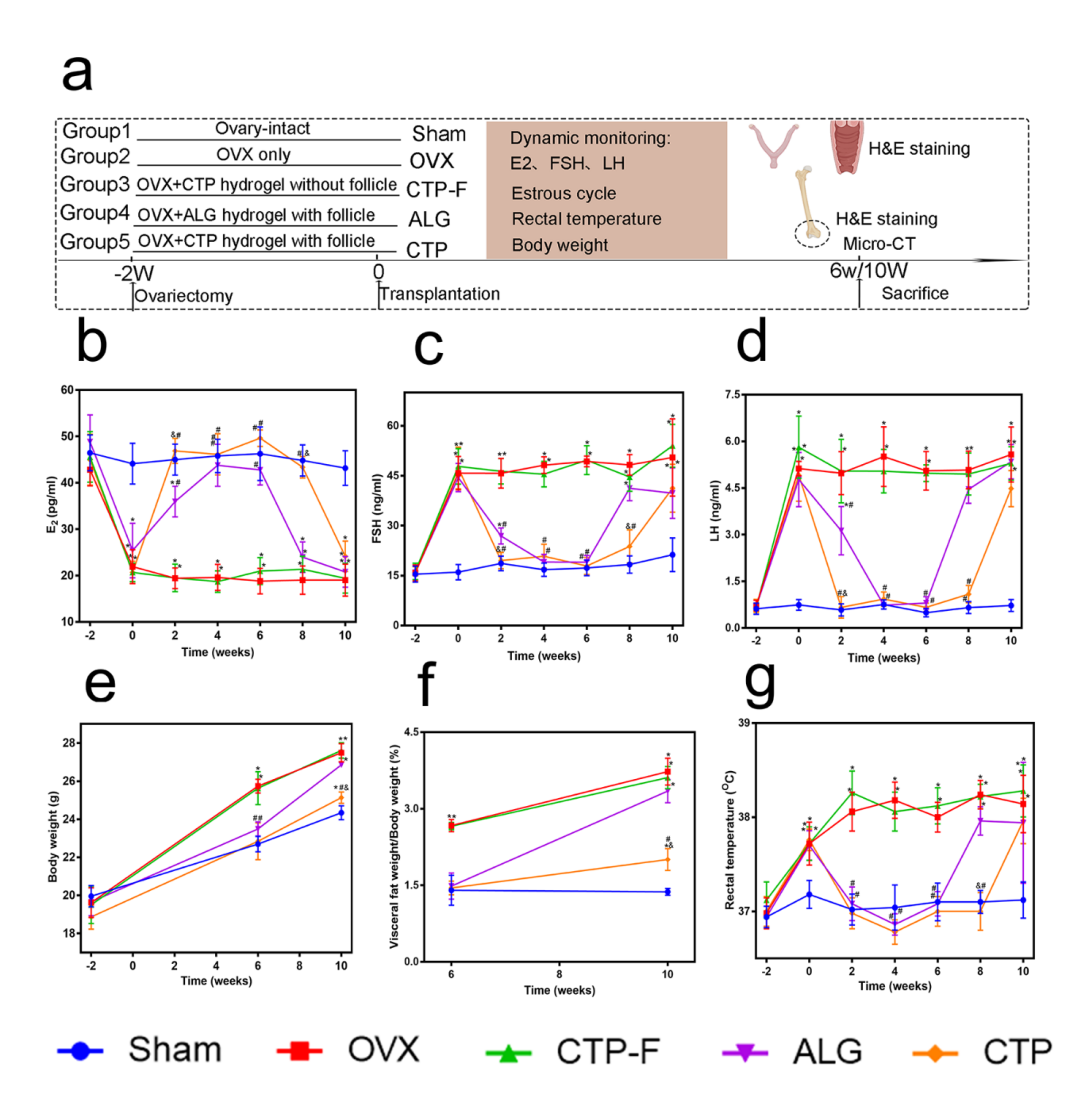
Hormone levels in the artificial ovaries and their effects on body composition. (a) Schematic illustration of the animal experiments. (b-d) Serum E_2_, FSH, and LH levels were measured twice a week for 10 weeks. (e) Body weight was measured at 6 and 10 weeks after transplantation. (f) Visceral fat was measured at 6 and 10 weeks after transplantation. (g) Rectal temperature was measured twice a week for 10 weeks. * indicates significance at P < 0.05 compared with the sham groups; # indicates significance at P < 0.05 compared with the OVX groups; $ indicates significance at P < 0.05 compared with the CTP-F groups; & indicates significance at P < 0.05 compared with the ALG groups, determined using one-way ANOVA, followed by Student–Newman–Keuls post-hoc analysis

After isolation, the follicles were encapsulated in CTP or ALG hydrogels to construct AOs and then transplanted into OVX mice to investigate whether AO could mimic the functions of natural ovaries. Figure 5a shows a schematic illustration of the experimental design. Serum levels of both follicle-stimulating hormone (FSH) and luteinizing hormone (LH) were elevated in OVX mice owing to the loss of the negative feedback control loop (Fig. 5c-d). CTP-F mice exhibited lower levels of serum E_2_ and higher levels of FSH and LH than the sham mice (Fig. 5b-d). These results indicated the successful construction of the OVX model. Transplantation with ALG and CTP grafts significantly increased the serum E_2_ levels and decreased the levels of serum FSH and LH compared with those in OVX and CTP-F mice (P < 0.05). The physiological levels of FSH, LH, and E_2_ were remarkably maintained until 8 weeks in the CTP groups, and the function of the CTP grafts was sustained for 2 weeks longer than that of the ALG grafts (Fig. 5b-d).

Oestrogen loss leads to body fat accumulation [36] and body weight [37] gain. Figure 5e-f depicts this phenomenon by comparing the sham and OVX groups. Six weeks after transplantation, mice in the OVX groups transplanted with the ALG and CTP grafts exhibited body weights comparable to those in the sham groups (Fig. 5e). Although the mean body weights of mice in the ALG and CTP groups were higher than those in the sham groups at 10 weeks after transplantation, the mean body weights of mice in the CTP groups were lower than those in the ALG groups (P < 0.05) (Fig. 5e). Moreover, the amount of visceral fat in mice in the CTP groups was lower than those in the ALG groups at 10 weeks after transplantation (P < 0.05) (Fig. 5f). Rectal temperature was significantly higher in OVX mice than in sham mice, suggesting that the OVX mice experienced hot flushes (P < 0.05) (Fig. 5g). Mice in the OVX groups transplanted with ALG and CTP grafts exhibited rectal temperatures comparable to those in the sham groups at 6 weeks after transplantation. However, the rectal temperatures of mice in the ALG groups were similar to those in the OVX groups at week 8, and the mice in the CTP and OVX groups showed similar rectal temperatures at week 10 (Fig. 5g). These results indicated that AOs constructed using ALG and CTP hydrogels could function normally in OVX mice and that ALG and CTP grafts completely prevented the increase in body weight and rectal temperature in OVX mice at 6 weeks after transplantation. The therapeutic effects of CTP grafts were notably superior to those of ALG grafts in OVX mice at 10 weeks after transplantation.

### Transplantation of AOs restored OVX-induced uterine atrophy

**Fig. 6:**
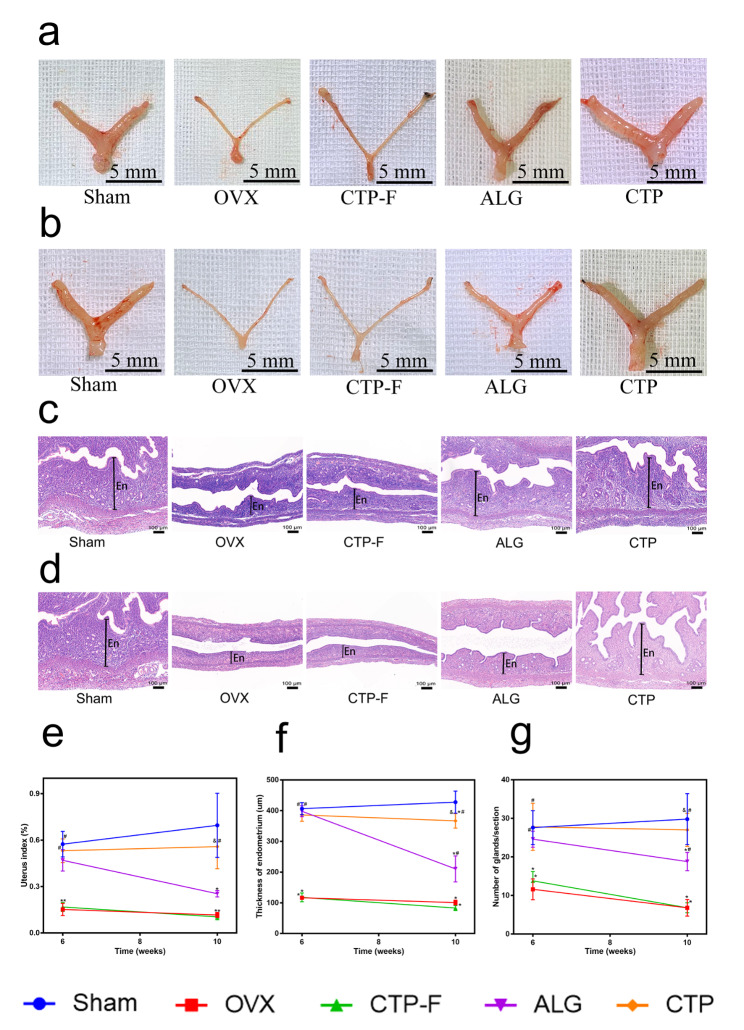
Effects of artificial ovaries transplantation on the uteri of ovariectomised (OVX) mice. (a) Representative images of the uterus at 6 weeks after transplantation. (b) Representative images of the uteri at 10 weeks after transplantation. (c) Images of the uterine sections stained with haematoxylin and eosin (H&E) at 6 weeks after transplantation (En, endometrium). (d) Images of the uteri stained with H&E at 10 weeks after transplantation (En, endometrium). (e) Uterine index after transplantation. (f) The thickness of the endometrium and (g) the number of glands in the uterus were calculated in each group after transplantation. The data are represented as the mean ± standard deviation. * indicates significance at P < 0.05 compared with the sham groups; # indicates significance at P < 0.05 compared with the OVX groups; $ indicates significance at P < 0.05 compared with the CTP-F groups; & indicates significance at P < 0.05 compared with the ALG groups, determined using one-way ANOVA, followed by Student–Newman–Keuls post-hoc analysis

The uterus begins to atrophy after losing oestrogen-induced protection. We observed that the uteri of OVX mice became thinner and longer than those of sham mice (Fig. 6a-b). However, the OVX mice transplanted with ALG and CTP grafts demonstrated general uterine morphologies similar to those of the sham mice at 6 and 10 weeks after transplantation. Figure 6e shows that the uterine indices of OVX and CTP-F mice were significantly lower than those of the sham mice (P < 0.05); however, the uterine indices of OVX mice transplanted with both ALG and CTP grafts did not differ from those of the sham mice at 6 weeks after transplantation (P > 0.05). Conversely, 10 weeks after transplantation, the CTP grafts resulted in uterine indices comparable to the values in the sham groups (P > 0.05), and the ALG grafts yielded comparable indices to those in the OVX groups (P > 0.05). Subsequently, we evaluated the histological changes in the uterus after transplantation. We observed that the endometrium became thinner, and the number of glands decreased in OVX and CTP-F mice compared with the sham mice (Fig. 6c-d). In contrast, the endometrial thickness increased and more glands appeared in the ALG and CTP groups, similar to the levels observed in the sham groups at 6 weeks after transplantation (Fig. 6f-g). At 10 weeks after transplantation, endometrial thickness and gland density of mice in the ALG groups were significantly different from those in the sham groups; however, these values were similar between mice in the CTP and sham groups (Fig. 6f-g). Our results demonstrated that CTP grafts improved uterine atrophy associated with oestrogen deficiency better than ALG grafts at 10 weeks after transplantation.

### AO transplantation restored OVX-induced vaginal atrophy

**Fig. 7:**
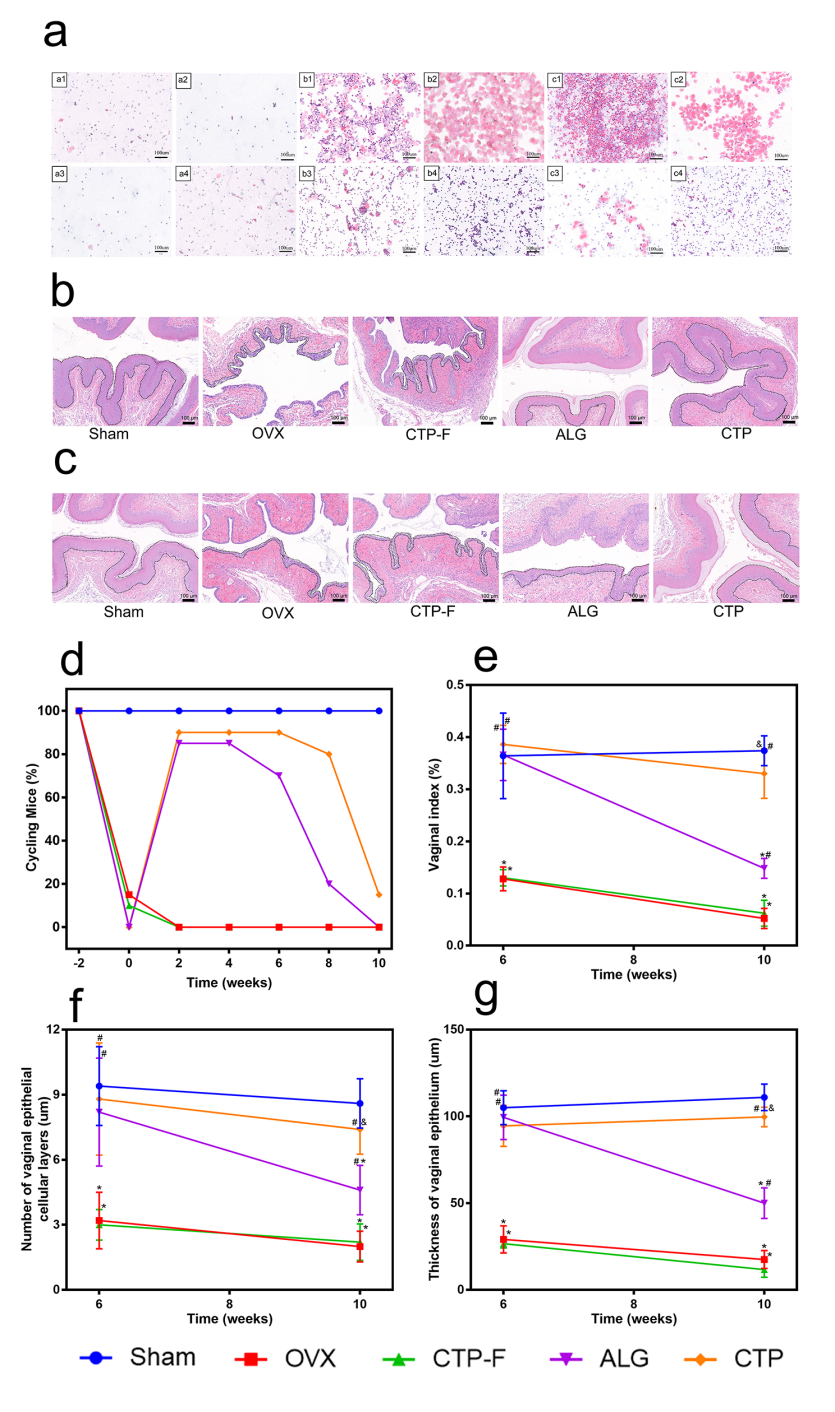
Effects of artificial ovaries transplantation on the vagina in ovariectomised (OVX) mice. (a) Images a1-a4, b1-b4, and c1-c4 depict representative cytology images of the OVX mice, ALG mice, and CTP mice, respectively. (b) Images of the vagina stained with haematoxylin and eosin (H&E) at 6 weeks after transplantation. (c) Images of the vagina stained with H&E at 10 weeks after transplantation. (d) Proportion of mice with oestrus cycles after transplantation. (e) Vaginal indices after transplantation. (f) The thickness of epithelial cells and (g) number of epithelial layers in the vagina were calculated for each group after transplantation. The data are represented as the mean ± standard deviation. * indicates significance at P < 0.05 compared with the sham groups; # indicates significance at P < 0.05 compared with the OVX groups; $ indicates significance at P < 0.05 compared with the CTP-F groups; & indicates significance at P < 0.05 compared with ALG, determined using one-way ANOVA, followed by Student–Newman–Keuls post-hoc analysis

The vagina begins to atrophy and lose mass after OVX [38]. The oestrus cycle in mice is generally categorised into four stages (proestrus, oestrus, metestrus, and dioestrus) [39]. The oestrus cycle of mice in the OVX and CTP-F groups was arrested at the metestrus/dioestrus stage; however, mice in the ALG and CTP groups exhibited dynamic and regular cycling even after transplantation (Fig. 7a). Moreover, 90% and 85% of the mice in the CTP and ALG groups, respectively, showed dynamic cycling at 6 weeks after transplantation (Fig. 7d). The number of mice with oestrus cycles decreased gradually over time, and no mice in the ALG groups experienced oestrus cycles; however, approximately 25% of the mice in the CTP groups experienced oestrus cycles at week 10 (Fig. 7d). Figure 7e shows that the vaginal indices of mice in the ALG groups were not different from those in the OVX groups; nevertheless, the vaginal indices were significantly different between the CTP and OVX groups at 10 weeks after transplantation (P < 0.05). Finally, the thicknesses of the vaginal epithelium and epithelial cellular layer were quantitatively analysed using H&E staining (Fig. 7b-c). OVX mice exhibited thinner vaginal epithelia and fewer vaginal epithelial cellular layers than sham mice (Fig. 7f-g). Conversely, compared with the vaginal epithelial or epithelial cellular layer thickness of mice in the sham groups, those in the ALG and CTP groups showed no significant differences at 6 weeks after transplantation (Fig. 7f-g). At 10 weeks after transplantation, the thicknesses of both vaginal epithelia and epithelial cellular layers of mice in the CTP groups were similar to those in the sham groups; however, these parameters significantly differed between mice in the ALG and sham groups (Fig. 7f-g).

### AO transplantation restored OVX-induced bone loss

**Fig. 8:**
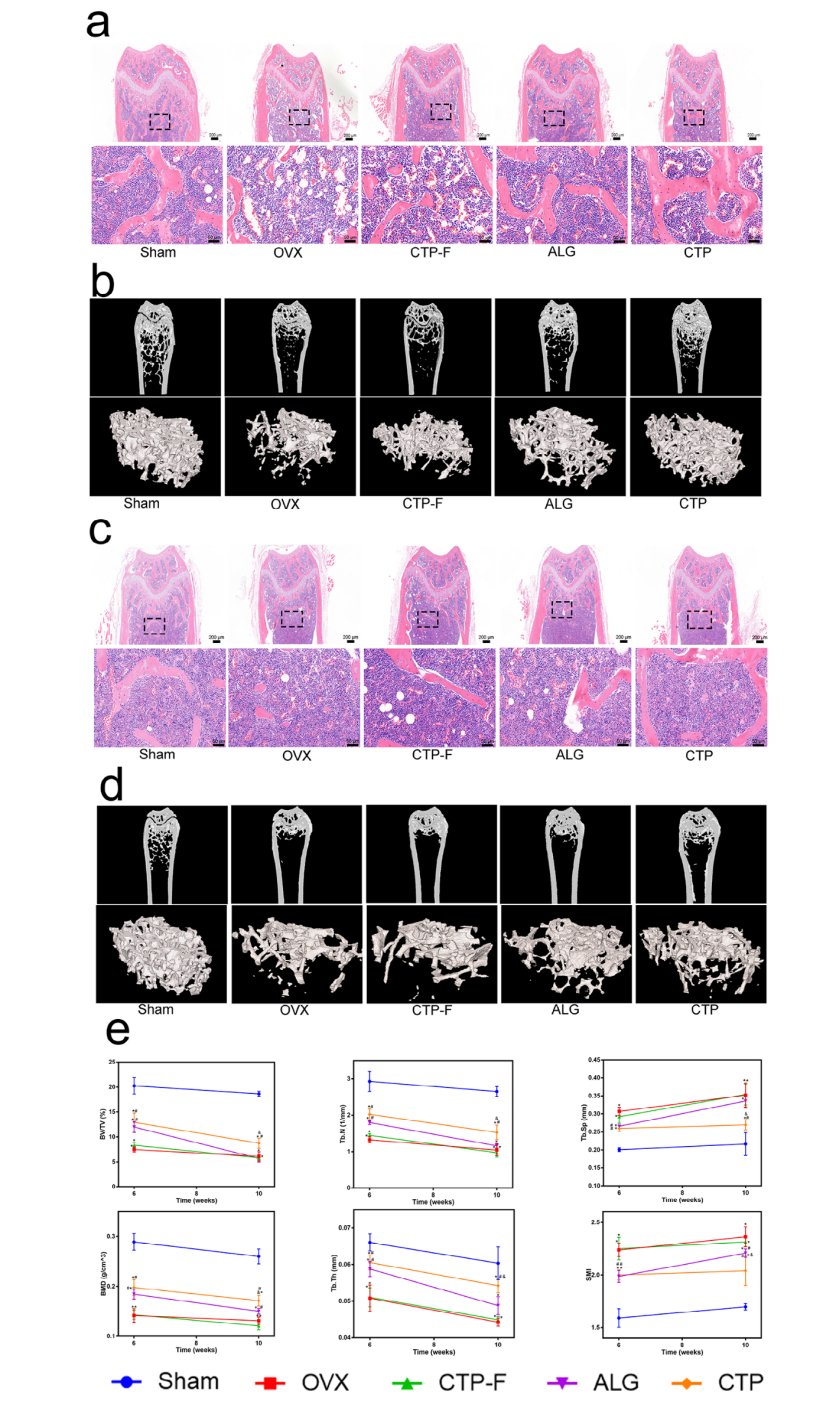
Artificial ovaries transplantation restored ovariectomised (OVX)-induced bone loss after transplantation. (a) Representative images of haematoxylin and eosin (H&E) staining and (b) micro-computed tomography (micro-CT) imaging analysis at 6 weeks after transplantation. (c) Representative images of H&E staining and (d) micro-CT imaging analysis at 10 weeks after transplantation. (e) Quantitative analyses of BV/TV%, Tb.Sp, Tb.N, Tb.Th, BMD, and SMI. The data are represented as the mean ± standard deviation. * indicates significance at P < 0.05 compared with the sham groups; # indicates significance at P < 0.05 compared with the OVX groups; $ indicates significance at P < 0.05 compared with the CTP-F groups; & indicates significance at P < 0.05 compared with ALG groups, determined using one-way ANOVA, followed by Student–Newman–Keuls post-hoc analysis

Oestrogen deficiency can lead to osteoporosis and fractures [40]. To examine the effects of AOs on the loss of bone mass in OVX mice, femurs were collected for micro-CT imaging and H&E staining (Fig. 8a-d). H&E staining revealed that the structure of the trabeculae was disordered, number of trabeculae decreased, and number of fat vacuoles increased in the bone marrows of mice in the OVX and CTP-F groups compared to those in the sham groups (Fig. 8a). Micro-CT imaging confirmed that bone mineral density (BMD), trabecular number (Tb.N), trabecular thickness (Tb.Th) and percent bone volume (BV/TV%) were significantly reduced, the trabecular separation (Tb.Sp) and structural model index (SMI) markedly increased in the OVX mice compared to sham mice (Fig. 8e). The ALG and CTP grafts notably alleviated bone loss in the distal femur of OVX mice; however, none of the treated groups exhibited bone loss restoration similar to the sham groups at 6 and 10 weeks after transplantation (Fig. 8e). At 10 weeks after transplantation, the parameters BV/TV%, Tb.N, Tb.Sp and Tb.Th in the ALG mice were not significantly different from those in the OVX mice; however, these parameters differed significantly between the ALG and the sham mice (P < 0.05). However, the BMD, Tb.N, Tb.Th and BV/TV% in the CTP mice were significantly different from those in the OVX and ALG mice, as shown in Fig. 8e. Conclusively, transplantation with AOs partially protects against OVX-induced osteoporosis, and the therapeutic effects of CTP grafts were found to be superior to those of ALG grafts at 10 weeks after transplantation.

### Degradability and biocompatibility of scaffolds in vivo

**Fig. 9:**
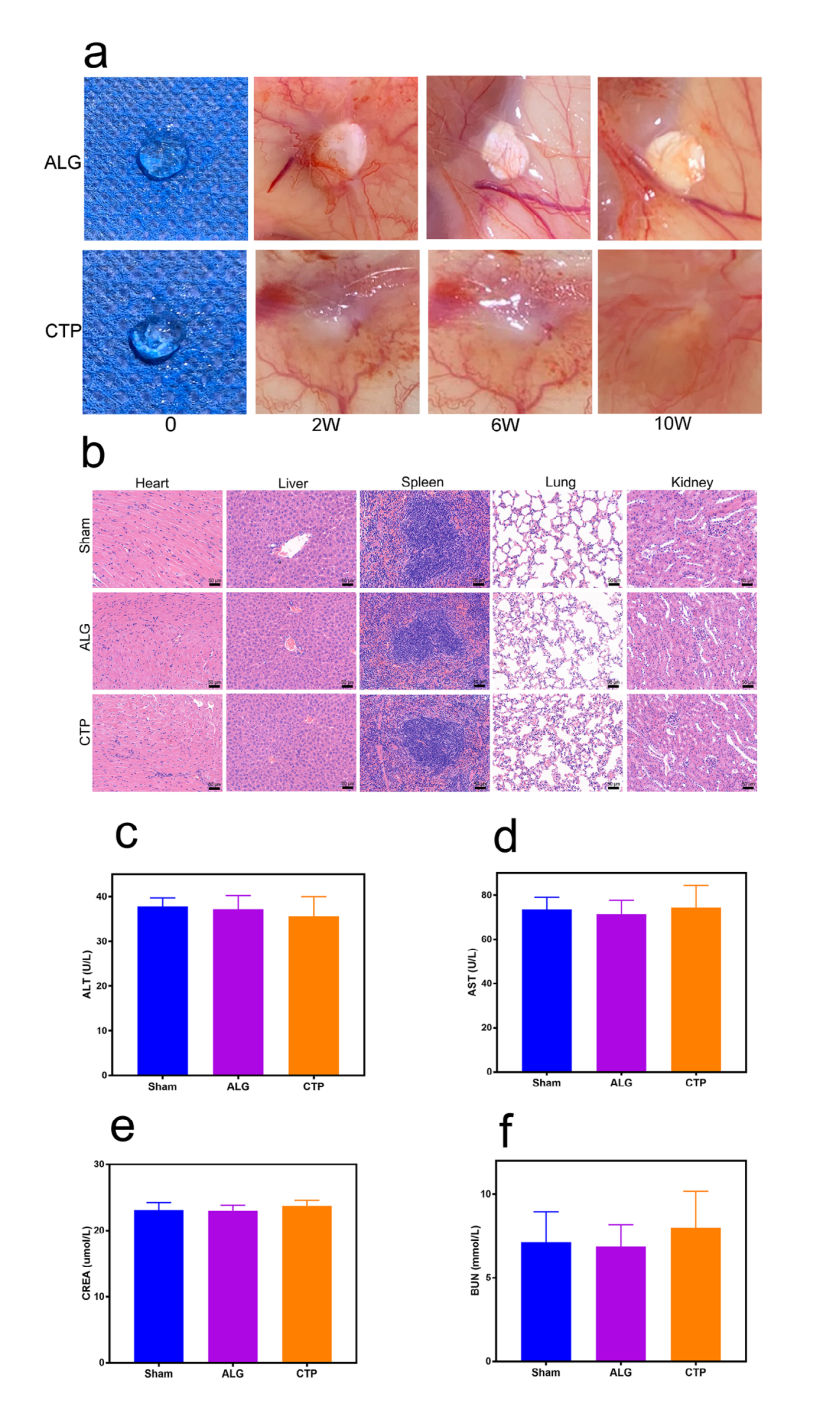
Degradability and biocompatibility of scaffolds in vivo. (a) Representative images of the grafts. (b) Representative images of haematoxylin and eosin staining of the heart, liver, spleen, lung, and kidney tissue sections from the CTP and ALG groups for biocompatibility validation in vivo at 10 weeks after transplantation. (c-f) Biochemical analyses of hepatic (ALT and AST) and kidney function (BUN and CREA) after implantation with hydrogels for 10 weeks

The biocompatibility of CTP hydrogels in vivo is crucial for its application in the fabrication of AOs. Figure 9a shows that the ALG hydrogels were non-degradable, consistent with previous data [12]. Conversely, the CTP hydrogels were degradable, consistent with the 3D-hydrogel criteria (Fig. 9a). Furthermore, we observed no evident signs of tissue damage or inflammatory lesions in any of the major organs (heart, liver, spleen, lung, and kidney) (Fig. 9b). Biochemical analyses of hepatic and kidney function revealed no significant differences between mice in the CTP and sham groups (P > 0.05) (Fig. 9c-f). Thus, the biocompatibility of the CTP hydrogels is satisfactory for its future biomedical applications.

## Discussion

Several types of scaffolds, such as collagen, fibrin, alginate, poly (ethylene glycol), and decellularised extracellular matrix have been designed and used as matrices for AO assembly [20]. This is the first study to show the survival and growth of isolated follicles in a chitin-based scaffold in vitro and in vivo. In vitro, CTP hydrogels not only demonstrate sufficient rigidity to maintain the 3D follicular structure but also support follicular growth for a longer duration than ALG hydrogels. In vivo, CTP grafts engineered with follicles effectively ameliorated bone loss, atrophy of the reproductive organs, and body composition in OVX mice; these CTP grafts showed superior therapeutic effects compared to those exhibited by ALG grafts.

The transition from a primordial follicle to a primary follicle in mice takes approximately one week, whereas a primary follicle transitions to a preovulatory follicle in approximately two weeks [41]. Our study found that early secondary follicles require 13 days to develop into preovulatory follicles, consistent with follicular growth under physiological conditions [42, 43]. At the end of the culture period, the follicles cultured in CTP hydrogels demonstrated higher survival rates, yielded higher oestrogen levels, and exhibited larger diametres than those cultured in ALG hydrogels (Fig. 2). The reduced oestradiol production in the ALG hydrogels compared to that observed in the CTP hydrogels may be attributed to the inadequate production of androgens by theca cells, aromatisation deficiency in GCs, or decreased proliferation of GCs. We speculate that the mechanical properties, such as the Young’s modulus and degradability, of the ALG hydrogels are inferior to those of the CTP hydrogels. Hence, we conclude that CTP hydrogels are more suitable for in vitro follicle growth than ALG hydrogels.

The formation of functional blood vessels during the early post-grafting period is beneficial to improve the follicular recovery rate. The follicle recovery rate after 1 week of grafting in the ALG matrix was similar to that of previous results (12%) [44]. Endothelial cells play an essential role in angiogenesis, which is a prerequisite after avascular grafting procedures. The numbers of CD34-positive vessels and Ki-67-positive cells in the CTP grafts were higher than those in ALG grafts at 1 week after transplantation, indicating that host endothelial cell infiltration and vascularisation in CTP grafts were superior to those in ALG grafts. Hence, the follicular recovery rate in CTP hydrogels (28%) was significantly higher than that in ALG hydrogels (17.2%) after 1 week of grafting. The follicular recovery rates (an average of 21%) varied with different matrices; therefore, a more effective method is required to reduce follicular loss [45]. We believe that the higher follicle recovery rate obtained in this study implies that chitin is a promising matrix for AO construction. Williams et al. [46] found that ALG hydrogels supplemented with 0.5% chitosan showed better angiogenic potential, with significantly increased blood vessel formation, compared with ALG hydrogels alone. The angiogenesis-promoting mechanism of chitosan involves the recruitment of vascular precursor cells by controlling the release of sphingosine-1-phosphate. In future studies, we will optimise our CTP hydrogels or load pro-angiogenic factors to further enhance vascularisation.

Approaches using cell-based hormone replacement therapy (cHRT) are feasible for hormone replacement, as they can restore the hypothalamus-pituitary-ovary (HPO) axis in a manner that cannot be accomplished via pHRT. In our study, follicles encapsulated in CTP hydrogels were implanted into the OVX mice to investigate whether the HPO axis could be restored by AOs. We found that serum oestradiol levels in CTP mice were restored to normal at 2 weeks after transplantation and were maintained until week 8 (Fig. 5b). The results for FSH and LH were notably consistent with those for oestradiol, confirming the restoration of the HPO axis (Fig. 5c-d). In contrast, serum oestradiol levels in the ALG mice were restored to normal at 4 weeks after transplantation and were maintained only until week 6; this observation is possibly because of low angiogenesis early after transplantation, resulting in a follicular loss in the ALG grafts. These results imply that the ALG grafts functioned slowly. Moreover, the non-degradability of the ALG hydrogels limited the growth of follicles, causing excessive follicular loss after transplantation; therefore, the duration of ALG grafts function was relatively short than the CTP grafts.

A previous study reported that the poly (ethylene glycol) vinyl sulfone hydrogel with encapsulated follicles successfully functioned as an AO for 60 days in vivo; however, the study did not investigate the effect of oestrogen on target organs [47]. Our study compared the effects of different grafts on the target organs. Similar to previous research, we observed significant vaginal and uterine atrophy in the OVX mice. At 10 weeks after transplantation, the vaginal indices in the ALG mice were significantly different from those in the sham mice, whereas the vaginal indices of the CTP and sham mice were similar. Endometrial thickness and the number of uterine glands improved at 6 weeks after transplantation in both the ALG and CTP mice; however, these parameters significantly differed between the ALG and sham mice at 10 weeks after transplantation (Fig. 6). These results showed that both the ALG and CTP grafts exhibited the same function of improving the uterine and vaginal indices at 6 weeks after transplantation; however, the CTP grafts yielded better results than ALG grafts at 10 weeks after transplantation. This observation possibly results from the formation of more new functional blood vessels in the early stage after transplantation, resulting in an increased follicular survival rate in mice of the CTP groups. Moreover, the CTP hydrogels were degradable and adapted to the volumetric expansion of the follicle (Fig. 9a).

Osteoporosis is a chronic bone metabolic disease characterised by low bone mass and deterioration of the bone microarchitecture, directly increasing the risk of fracture. Therefore, we investigated the effects of AO transplantation on OVX-induced osteoporosis in mice. Figure 8 demonstrates the quantification of the bone volume of the control animals (ovary-intact or OVX) compared with that of mice transplanted with cHRT-bioengineered ovarian construct. Our results revealed that the bone trabecular structure improved in mice that received ALG and CTP grafts compared to that of OVX mice at 6 weeks after transplantation; however, none of the mice that received grafts could restore bone structures to similar levels exhibited by ovary-intact mice. Importantly, the therapeutic effect of CTP grafts was superior to that of ALG grafts at 10 weeks after transplantation. A previous study showed that apart from oestrogens, the HPO axis reproductive hormones play a crucial role in modulating key processes in skeletal homeostasis [48]. Moreover, studies in post-menopausal women have demonstrated that a decrease in oestrogen results in reduced expression of inhibin B and inhibin A and increased serum FSH levels, ultimately leading to considerable bone loss [49]. Activin and inhibin levels were not detected in this study. This treatment might be too late to reverse oestrogen deficiency-induced bone loss. In future studies, we will aim to transplant AOs immediately after ovariectomy, instead of waiting for 14 days, to observe whether the quantitative bone parameters are restored to normal levels.

AOs, as an emerging HRT, may be used to restore steroid hormones in many clinical scenarios. AOs engineered with autologous follicles could be a safer approach to restore ovarian function and avoid the risk of reintroducing malignant cells in cancer patients. Moreover, it is possible to transplant AOs for post-menopausal women with solid organ transplantation needs, and without additional immunosuppressants. However, in this study, we conducted experiments only in mice, and clinical studies to validate these results in humans are warranted in the future.

## Conclusion

Our study is the first to demonstrate that CTP hydrogels could support folliculogenesis for a longer duration than ALG hydrogels both in vitro and in vivo. In vitro, CTP hydrogels could support follicular growth better than ALG hydrogels, as they can better mimic the ovarian microenvironment. In vivo, the therapeutic effects of CTP grafts were superior to those of ALG grafts at 10 weeks after transplantation. After transplantation, CTP hydrogels could promote early angiogenesis, better mimic the ovarian microenvironment, and later adapt to follicular volume expansion owing to their biodegradability. The findings presented in this study can help in the development of CTP hydrogel-based AOs, with potential applications in the clinical treatment of menopausal symptoms. However, the aforementioned results warrant a long-term study and further validation in large-scale animal experiments.

## Electronic supplementary material

Below is the link to the electronic supplementary material.

Supplementary Material 1

## Data Availability

Not applicable.

## Abbreviations

AO: Artificial ovary
ALG: Alginate
OVX: Ovariectomised
GCs: Granulosa cells
pHRT: Pharmacologic hormone replacement therapy
cHRT: Cell-based hormone replacement therapy
IVC: In vitro culture
HPO: Hypothalamus-pituitary-ovary
FSH: Follicle-stimulating hormone
LH: Luteinizing hormone
H&E: Hematoxylin and eosin
AOD: Average optical density
TUNEL: Terminal deoxynucleotidyl deoxyuridine triphosphate nickend labeling
BMD: Bone mineral density
BV/TV%: percent bone volume
Tb.N: Trabecular number
Tb.Th: Trabecular thickness
Tb.Sp: Trabecular separation
SMI: Structural model index
