# Supplementary material for: Artificial ovaries constructed from biodegradable chitin-based hydrogels with the ability to restore ovarian endocrine function and alleviate osteoporosis in ovariectomized mice
Source: Reprod Biol Endocrinol. 2023 May 19;21:49. doi: 10.1186/s12958-023-01092-8 (PMC10197240; doi:10.1186/s12958-023-01092-8)
Supplement: Supplementary file 1 — Supplementary Material 1 [file 12958_2023_1092_MOESM1_ESM.docx]

**SUPPLEMENTARY DATA
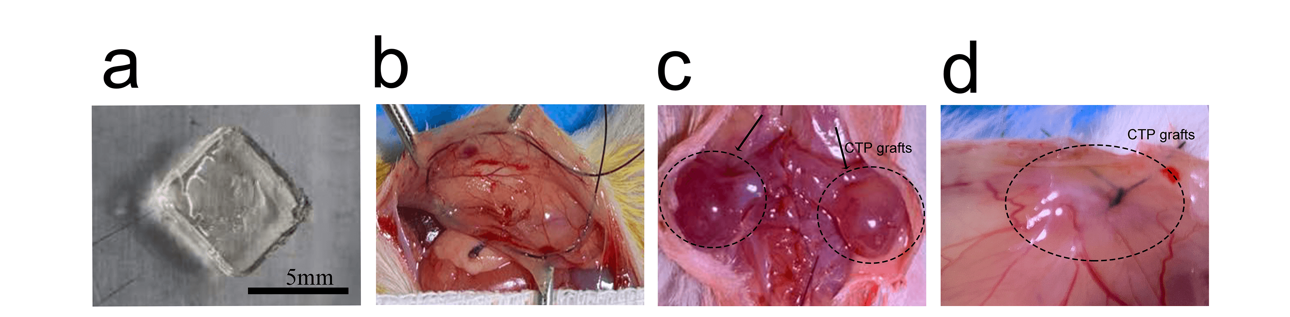
**

**Fig. S1.** The process of transplantation. (a) AOs constructed using CTP hydrogels. (b) Grafts were transplanted into the peritoneal pockets. (c) AOs inside the peritoneal pockets after grafting. (d) CTP grafts inside the peritoneal pockets after 10 weeks of grafting.
